# earEOG via periauricular electrodes to facilitate eye tracking in a natural headphone form factor

**DOI:** 10.1038/s41598-025-16839-z

**Published:** 2025-09-12

**Authors:** Tobias King, Michael Knierim, Philipp Lepold, Christopher Clarke, Hans Gellersen, Michael Beigl, Tobias Röddiger

**Affiliations:** 1 Karlsruhe Institute of Technology, TECO/Pervasive Computing Systems, 76131 Karlsruhe, Germany; 2 Karlsruhe Institute of Technology, Institute of Information Systems and Marketing, 76131 Karlsruhe, Germany; 3 University of Bath, Human Computer Interaction, Bath, BA2 7AY UK; 4 Lancaster University, Interactive Systems, Lancaster, LA1 4YW UK; 5 Department of Computer Science, Aarhus University, 8200 Aarhus, Denmark

**Keywords:** Information technology, Data processing, Machine learning

## Abstract

Eye tracking technology is frequently utilized to diagnose eye and neurological disorders, assess sleep and fatigue, study human visual perception, and enable novel gaze-based interaction methods. However, traditional eye tracking methodologies are constrained by bespoke hardware that is often cumbersome to wear, complex to apply, and demands substantial computational resources. To overcome these limitations, we investigated the application of Electrooculography (EOG) eye tracking using 14 electrodes positioned around the ears, integrated into a custom-built headphone form factor device. In a controlled laboratory experiment, 16 participants tracked a series of on-screen stimuli designed to induce smooth pursuits and saccades. Data analysis identified the optimal electrode pairs for tracking vertical and horizontal eye movements, benchmarked against gold-standard EOG and camera-based eye tracking. The electrode montage closest to the eyes provided the best results for horizontal eye movements. One-dimensional smooth pursuit eye movements measured via earEOG exhibited a high correlation with the gold-standard for horizontal 1D pursuits spanning $2.5^{\circ }$ to $15^{\circ }$ visual angle for the best performing electrode pair (${\textrm{r}}_{\textrm{EOG}} = 0.81, {\textrm{p}}=0.01$; ${\textrm{r}}_{\textrm{CAM}} = 0.56, {\textrm{p}}=0.02$). Vertical 1D smooth pursuits were only weakly correlated for the best performing pair (${\textrm{r}}_{\textrm{EOG}} = 0.32, {\textrm{p}}=0.03$; ${\textrm{r}}_{\textrm{CAM}} = 0.31, {\textrm{p}}=0.05$). Voltage deflections of earEOG and gold-standard EOG for saccades from $2.5^{\circ }$ to $15^{\circ }$ in the four cardinal directions are highly correlated for horizontal eye movement (${\textrm{r}}_{\textrm{left}} = 0.99, {\textrm{p}}<0.001$; ${\textrm{r}}_{\textrm{right}} = 1.0, p<0.001$) but not for vertical eye movements (${\textrm{r}}_{\textrm{up}} = 0.79, {\textrm{p}}=0.06$; ${\textrm{r}}_{\textrm{down}} = 0.35, {\textrm{p}}=0.53$). A regression model was employed to predict absolute gaze angle changes of horizontal saccades using earEOG and gold-standard EOG. In the left and right directions, the earEOG model achieved a mean absolute angular error of $3.99 ^\circ \pm 3.45 ^\circ$, for saccades ranging from $2.5^{\circ }$ to $15^{\circ }$. In comparison, gold-standard EOG attained mean absolute angular error of $2.98 ^\circ \pm 2.44 ^\circ$. Overall, horizontal earEOG demonstrated strong performance, indicating its potential effectiveness in our setup. On the other hand, vertical earEOG showed significantly poorer results, suggesting that it may not be feasible with our current configuration.

## Introduction

Eye tracking is a widely employed technique for sensing and interaction that typically involves either camera-based^1,2^ or Electrooculography (EOG)^2–4^ methods. While camera-based eye tracking can be precise, it is computationally intensive and requires a significant amount of power^2^. On the other hand, traditional EOG is less computationally demanding and even works when the eyes are closed. However, it is restricted to tracking relative changes in gaze direction, is subject to signal drift and is relatively invasive as electrodes have to be glued on the face around the eyes. Despite these limitations, EOG has various interesting applications. In a medical context, EOG can be applied in sleep studies^5^ or to diagnose balance disorders^6^. Outside the clinic, EOG can be used for hands-free interaction with wearable devices^3,7,8^, to detect cognitive load^9^, to classify user activities^4^, to quantify reading activity up to word-level accuracy^10,11^, and for providing directed auditory attention in noisy environments^12–14^.

To make EOG more feasible, past work implemented electrodes into smart glasses which improves wearability and more naturally integrates into the everyday-life of the user^3,11^. Prior research has also suggested that eye tracking using electrodes placed inside the ear canal^13–17^ or at the mandible^18^ is generally feasible. Favre-Felix et al.^14^ investigated the use of ear-based EOG and motion sensors around the ear to estimate absolute horizontal eye gaze in multi-talker situations, showing promising results when the head was fixed. However, hardware issues hindered reliable estimations when the head was free. Manabe et al.^17^ developed an earphone-based eye gesture input interface using conductive rubber electrodes. In another paper, the same authors^18^ proposed a headphone-type gaze detector with electrodes placed at the mandible on one ear to estimate gaze direction. Based on experiments with a single user, they achieve an estimation error of $4.4^{\circ }$ (horizontal) and $8.3^{\circ }$ (vertical) in a 5 $\times$ 3 fixation point grid ($20^{\circ }$ visual angle between fixation points) and lay the foundation for our work. Similar to smart glasses, ear-based devices, such as the OpenEarable and OpenEarable EXG platforms^15,19^, have the potential to be more comfortable, discreet, and portable than traditional EOG. Furthermore, the ear is an ideal location for integrating eye tracking with audio applications for example for directed auditory attention in noisy environments and with hearing aids^13,14,16^.

The effectiveness of in-ear-based eye tracking for horizontal eye movements was explored in related work^13,14,16,17^. However, incorporating eye-tracking capabilities into headphones could have several advantages over the in-ear EOG method that we explore in this paper. Firstly, the proximity of headphones to the eyes enhances the sensitivity to eye movements, leading to potentially more accurate measurements of changes in eye position. To this extent, we seek to expand upon the initial work of Manabe et al.^18^ and evaluate headphone-based eye tracking in-depth with a large number of participants.

**Fig. 1:**
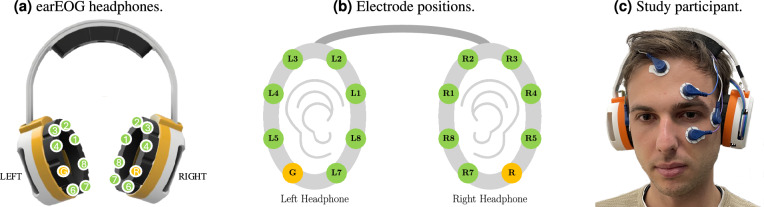
Overview of earEOG headphones showing the electrode positions and study apparatus. (**a**) The EOG headphones have a standard headphone form factor with 8 gold-plated electrodes (copper base) on each ear (One reference (R) and one Ground (G) electrode). The left headphone includes the Cyton Board while the right headphone includes the battery; (**b**) Schematic drawing showing the positioning of the electrodes around the ears; (**c**) A study participant wearing the EOG headphones and gold-standard Electrooculography simultaneously.

We thoroughly investigate the hypothesis that EOG-based eye tracking using electrodes placed around the ears in a regular headphone form factor is a reliable and accurate method for studying eye movements. To understand the achievable performance and add context, we ground our research in comparison to gold-standard EOG and camera-based eye tracking data. For our evaluation, a specialized headphone device was developed with 14 electrodes positioned strategically around the ears, see Fig. 1. Using the earEOG headphones, we conducted a lab study with 16 participants to collect data of two tasks.

For the first task, participants were asked to follow one-dimensional moving targets to elicit smooth pursuit eye motions^20^. Smooth pursuits are continuous eye movements that allow the eyes to follow a moving target smoothly without any jerks or abrupt changes in direction^21^. Smooth pursuits provide a continuous signal that can be more easily correlated with the changes in eye position than abrupt saccades, enabling a better analysis of the relationship between electrode signals and eye movements. This, in turn, helps us to determine the most effective electrode positions for capturing the nuances of eye movement and to evaluate the overall feasibility of the earEOG method. For horizontal eye movements, the bipolar montage of L8-R8 (difference in electrical signals between the two electrodes) yielded the highest correlation to horizontal gold-standard EOG. For vertical eye movements, only the montage of R3-R7 had significant but very weak correlation.

For the second task, participants were instructed to follow a point that jumped from the center of the screen at $0^{\circ }$ to $2.5^{\circ }$ up to $15^{\circ }$ in the four cardinal directions at $2.5^{\circ }$ increments. Using the ideal electrode positions identified from the previous analysis it was found that voltage deflections during saccades of earEOG and gold-standard EOG across all angles are mostly highly correlated for horizontal saccades. Vertical saccades were not significantly correlated to gold-standard EOG voltage deflections.

Building upon the relationship between voltage deflections and the underlying gaze angle, a regressions model was evaluated to calculate the absolute saccade angle from earEOG for the horizontal direction. On average, horizontal earEOG achieved an absolute angular accuracy of $3.99 ^{\circ }$ $\pm ~3.45 ^{\circ }$. In comparison, a similar model fitted on gold-standard horizontal EOG data achieves an absolute angular accuracy of $2.98 ^{\circ }$ $\pm ~2.44 ^{\circ }$. These findings demonstrate that earEOG is a promising method for eye tracking on the horizontal axis, showing good correlation with gold-standard EOG, which indicates its potential usability. Moreover, it could be a good approach for easily integrable, user-friendly electrodes in everyday life.

In sum, our contributions are: (i) a thorough investigation of EOG-based eye tracking using electrodes placed around the ears in a custom-built headphone form factor, providing earEOG—a novel approach to on-the-go wearable eye tracking in headphones; (ii) an evaluation of different electrode positions for earEOG and recommendations for the optimal placement of electrodes, to enable a more effective use of earEOG for eye tracking, thereby enabling a wider range of applications; and (iii) an evaluation of earEOG to predict absolute gaze angles in comparison to gold-standard EOG.

## Methods

### Participants

The participants in this study were recruited through a sample of convenience. Demographic information was self-reported by participants using questionnaires. Participants without sufficient vision capabilities, as determined by the need to wear glasses, were excluded from the study. This decision was made for two reasons: firstly, spectacles can interfere with the accuracy of the Tobii eye tracker, and secondly, they can physically obstruct the placement and contact of the earEOG electrodes. The study was conducted in a controlled environment to minimize the influence of external factors (45% humidity, 21$^{\circ }$C room temperature). No windows were present in the testing environment, and the brightness was measured to be 285 Lux using a Lutron LX-101 LUX-meter, with the sensor placed at face level and facing the monitor.

The study protocol was approved by the institutional review board of the Karlsruhe Institute of Technology (Germany) and followed all relevant ethical regulations in accordance with the Declaration of Helsinki. Before beginning the study, participants were informed of the study protocol, the purpose of data collection, and the specific data that would be collected. Informed consent was obtained from participants through the signing of an agreement form. Participants were not compensated.

### Apparatus

A 14-channel electric potential data collection setup was implemented using an OpenBCI Cyton + Daisy biosensing board, with 7 channels around each ear. Additionally, the device included one ground electrode and one reference electrode. Vertical and horizontal gold-standard EOG was also acquired using the OpenBCI board via four standard Ag/AgCl gel sticky electrodes glued around the eyes, see Fig. 1a–c (Informed consent to publish the identifiable images was obtained from all the participants). The positions for the gold-standard electrodes were chosen based on established findings in the literature^16,18,22^. The OpenBCI was housed in a 3D-printed headphone enclosure. The gold-plated (copper base) electrodes around each ear are custom-built flex-PCBs that were fit to the headphone form factor. Such electrodes are also common in existing wearable EEG headsets (e.g., Open-cEEGrid^23^). Before application, the area around the ears was cleaned with isopropyl alcohol. All EOG data was sampled at 125 Hz. In addition, a stationary camera-based eye tracker (Tobii eyeX Pro) was employed to record ground-truth gaze angles at 60 Hz.

For data collection, a web-based tool was implemented that provides instructions to the participants and shows on-screen stimuli to elicit specific eye movement patterns. The experiment was conducted using a 23” monitor (1920px $\times$ 1080px) with a viewing distance of 50 cm. Participants were seated centered in front of the screen, with the vertical center of the screen positioned at eye level using a height-adjustable desk. This ensured that the visual stimuli could be presented at the desired size and angle in relation to the participants head and the screen size. The participant’s head position was not fixed in space, but the person conducting the study carefully monitored the head position and distance to the screen to intervene if participants had moved significantly during data collection.

#### Data collection procedure

After arriving at the lab, signing the consent forms, and asking any possible questions, participants were fitted with the earEOG headphones as well as gold-standard vertical and horizontal EOG electrodes. Participants then spent 10–15 min in this environment before the recording while the electrodes were prepared, which effectively controlled for light adaptation prior to testing^24^. They were then seated in front of the screen. The stationary eye tracker was calibrated using the built-in 9-point calibration procedure. Participants then followed on-screen instructions to complete the eye-based tasks for data collection. All tasks took approximately 7 min to complete and were repeated three times, totalling approximately 25 min of experiment duration per participant. Between each task, participants had a 10 s resting period and between each cycle, participants could rest their eyes freely for one minute. The order of tasks was not counterbalanced.

### Task, stimuli, and procedure

Participants were presented with two different tasks to collect two types of eye movements: smooth pursuits and saccades. Smooth pursuits were added to find the best electrode for vertical and horizontal eye movement tracking, respectively. Saccades were added as they are fundamental for many research studies, and the absolute angle is the most characteristic to understand eye movements.

#### Smooth pursuit task

**Fig. 2:**
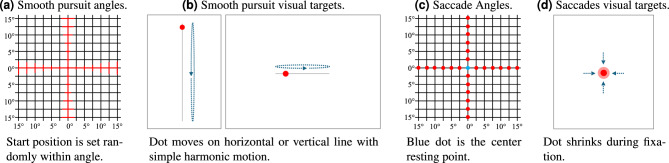
Overview of the different gaze stimuli and tasks presented to study participants. (**a**) 1D smooth pursuit points in horizontal and vertical directions with opening angles of $2.5^{\circ }$ to $15^{\circ }$. The dot moves on a circle around the center, with its starting position randomly selected on the circle; (**b**) visual target moving on a straight line with simple harmonic motion that is shown to participants to elicit smooth pursuit eye motion; (**c**) fixations points in four cardinal directions with $2.5^{\circ }$ increments and center resting point; (**d**) visual target point shown to participants that shrinks during fixation.

Smooth pursuit eye movements are continuous eye movements that follow a moving object, and they exhibit much slower characteristics compared to rapid eye movements such as saccades that shift the gaze from one object or point in space to another. The smooth pursuit task consisted of 1D smooth pursuits angles in both vertical and horizontal directions, see Fig. 2a. Participants were instructed to follow the gaze target that moved within a 2.5 to 15$^\circ$ visual opening angle from the center for 6 seconds each. The “opening angle” defines the maximum visual angle deviation from the center along the 1D movement axis. The frequency of the gaze target movement was set to 0.33, 0.5, and 1 Hz. The motion of the gaze target along its movement axis was modulated using a sine function, i.e., its position over time followed $\sin (x)$, resulting in smooth, continuous oscillation characteristic of simple harmonic motion, see Fig. 2b.

The gaze target had a diameter of 30 pixels, which is equivalent to 7.8 mm. The purpose of this task was to find the best electrode for vertical and horizontal eye movement tracking. The eye movement data collected during the smooth pursuit task will be correlated with the eye movements of the gold-standard EOG and camera-based gaze signals.

#### Saccade task

In the saccade task, participants were presented with a series of fixation points (Fig. 2c) to elicit saccade eye motion. Participants started with resting their gaze in the resting position at the screen’s center ($0^{\circ }$ visual angle in the x and y direction). The fixation point then jumped to 2.5-degree increments in each of the four cardinal directions (left, right, up, down) from 2.5 to 15$^\circ$. Each fixation point was presented for 2 s and shrank from 30 to 20 pixels (equivalent to 7.8 to 5.2 mm), see Fig. 2d. The target shrinks as this concentrates the focus of the user to the middle of the point and is a common procedure during eye tracker calibration, also for the Tobii eye tracker. After completing an angle, the fixation point returned to the center resting point for 2 s. The purpose of the saccade task was to collect saccade eye movement data to understand the relationship between earEOG signal deflections, gold-standard EOG, and absolute gaze angle changes.

### Data analysis

#### Preprocessing

The collected gold-standard EOG and earEOG data per ear are already aligned as both were sampled using the same device. For both the gold-standard EOG and the earEOG data, the signals for each channel were bandpass filtered between 0.1 and 15 Hz using a 5th-order Butterworth filter to eliminate noise and other artifacts, similar to methodologies employed in previous studies^25,26^. These pre-processing steps were carried out to ensure that the data was suitable for subsequent analysis.

#### Smooth pursuit analysis

In order to find the best electrode positions for measuring eye movements with electric potentials from around the ears, we used the ground truth gold-standard EOG as well as the camera-based eye tracker data and correlated them with differential earEOG electrode signals. We utilized the 1D smooth pursuit data (vertical and horizontal) to compute the best electrodes. Smooth pursuit eye movements were chosen because they exhibit continuous electric signal changes compared to rapid saccades, which create a sharp, short spike in the EOG signal. Consequently, smooth pursuits are less prone to artefacts and noise and allow for a continuous sequence to be correlated between the gold-standard and ear-based EOG principle, which improves the reliability of our results.

**Fig. 3:**
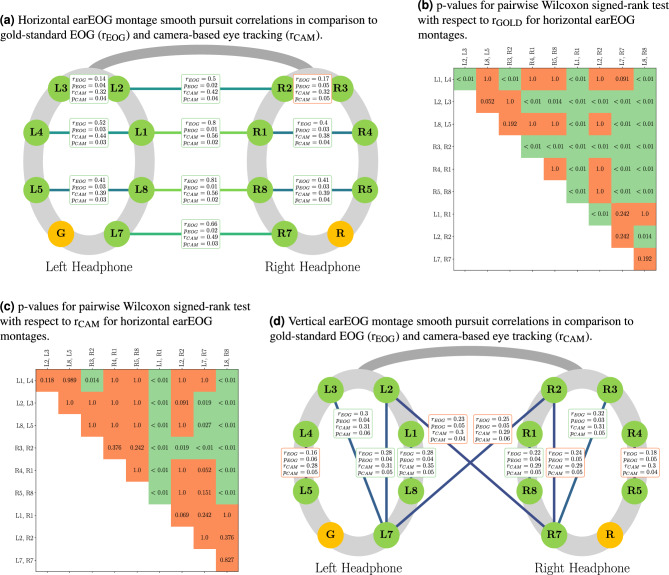
Comparison of earEOG electrode montages to gold-standard EOG and camera-based eye tracking. Measurement electrodes are shown in green color, while the ground electrode (G) and the reference electrode (R) are shown in gold. (**a**,**d**) The lines connect electrode pairs used as differential pairs to compute correlations with the gold-standard EOG ($r_{EOG}$) and Tobii eye-tracker ($r_{CAM}$) shown in the corresponding boxes. Line colors indicate how strong the correlation of the electrode pair is with respect to the gold-standard EOG. (**a**) Correlations of pairwise electrode montages with gold-standard horizontal EOG revealing that electrodes closer to the ears and at eye level yield higher correlations than electrodes on one ear or farther away from eye level; (**b**) Statistical tests showing p-values for pairwise Wilcoxon signed-rank tests with respect to gold-standard EOG for horizontal montages; (**c**) Statistical tests showing p-values for pairwise Wilcoxon signed-rank tests with respect to the eye tracking data for horizontal montages. (**d**) Correlations of pairwise electrode montages with gold-standard vEOG;

For horizontal eye movements, we considered electrode combinations which were aligned with the horizontal axis of the head (see Fig. 3a). Similarly, for vertical eye movements, we analyzed a subset of electrode pairs, specifically focusing on those positioned vertically above one another (see Fig. 3d). For all recorded smooth pursuits, the entire six-second sample was used for correlation. To account for signal propagation delays, we permitted a maximum lag of 12 samples ($\approx 100\,\textrm{ms}$) when calculating correlations between EOG channels. We also allowed up to 64 samples ($\approx 500\,\textrm{ms}$) for correlations between EOG and the eye tracker. This temporal mismatch is due to several factors: the mismatch of clocks between the two devices and the data processing lag which includes wireless communication for the OpenBCI. To correlate the data, we first preprocess the EOG and eye tracker data. For each saccade in the EOG data, we detrend the signal, apply a mean filter with filter length 50 and normalize the data to be in the interval [− 1, 1]. For each saccade as recorded by the eye tracker, we interpolate its values as some may be missing. Then, we resample the data to match the sampling rate of the EOG data (125 Hz), apply a butterworth bandpass filter of order 5 between 0.1 Hz and 15 Hz, apply a mean filter with filter length 50 and finally normalize the data to be in the interval [− 1, 1]. To compute the mean over the correlation coefficients, we first apply the Fisher z-transformation. The results are based on all sampled smooth pursuit speeds and angles across all observations from all participants. To identify the best vertical electrodes, we only used the vertical smooth pursuit data, and for the horizontal electrodes, we only use horizontal smooth pursuit data.

To identify whether the correlations between electrode montages and the gold-standard differ significantly, we employ a Friedman test. We compare each direction (horizontal and vertical) separately and use the gold-standard EOG as well as the camera-based eye tracker data as ground truth.

If the p-value of the Friedman test is below 0.05, we conduct a Wilcoxon signed-rank test (p-values are Bonferroni-corrected) to assess pairwise differences in the correlation values of each electrode pair with the gold-standard or the eye tracker.

#### Saccade analysis

Based on the ideal ear electrodes, obtained from the smooth pursuit analysis, we perform further analyses on the saccade data: (i) We analyse the average saccade signal for each direction and visual angle. (ii) We analyse the average voltage deflection for each direction and visual angle. (iii) We predict the visual angle of a saccade from the voltage deflection using a linear regression model. To perform these tasks, we label the start and end of the 576 saccades based on the gold-standard EOG signals in the direction of the saccade. In addition, we exclude saccades, for which no clear start and end could be identified. This leaves us with 565 saccades we use for further analysis.

Average saccade signal analysis: For each of the four directions and visual angles ($2.5^{\circ }$, $5^{\circ }$, $7.5^{\circ }$, $10^{\circ }$, $12.5^{\circ }$ and $15^{\circ }$), we compute the average signal of the saccades. This allows us to gain a more comprehensive understanding of the eye movement patterns and the corresponding electrical signals for both the gold-standard EOG and earEOG saccades. Saccade signals were subsequently averaged across both within- and between-observer replications.

Saccade amplitudes and corresponding EOG voltage deflections: In order to understand the relationship between the saccade amplitude and the voltage deflection in the earEOG signals, we calculate the mean voltage deflection for each direction and visual angle. This further allows us to study the relationship between the saccade amplitude and the voltage deflection in the earEOG signals in comparison to the gold-standard EOG signals. For each relevant saccade, the voltage deflection is determined by subtracting the mean of the last ten samples of the saccade from the mean of the first ten samples.

Saccade angle prediction: The measurement of voltage deflections through electrooculography (EOG) does not directly provide information on the absolute gaze angle. To overcome this limitation, we developed regression models that predict the horizontal gaze angle from earEOG or gold-startard EOG data when users were performing saccades as seen in Fig. 2c. The 565 valid saccades were all interpolated to contain the same number of data points and were assigned to the respective eye tracker ground truth gaze angle change. We then computed the voltage deflection by subtracting the mean of the last ten samples in a saccade recording from the mean of the first ten, noting that the actual saccade occurs approximately at the center of the recording. Using the *scikit-learn* library we trained a linear regression model to predict the change in gaze angle based on the optimal electrode pairing, obtained from the smooth pursuit analysis.

## Results

In total, 18 participants were recruited for the study. Because of data corruption issues, two participants had to be excluded from the study. Therefore, data analysis was conducted using data from 16 participants (11 male, 5 female). The mean age was 24.69 years (SD = 5.25 years, range 20 to 38 years). Participants were from European (N=12), Asian (N=3) and Latin-American (N=1) descent. 12 participants indicating no beard, while 2 had a beard, and another 2 had barely any beard. Regarding hair length, 8 participants had short hair, 6 had long hair, and 2 had medium-length hair. The mean inside distance from eye to eye was 3.16 cm (SD = 0.54 cm). The mean outside distance from eye to eye was 10.53 cm (SD = 0.5 cm). The mean inside distance from the left eye to the vertical center of the frontal left earEOG electrodes was 5.22 cm (SD = 0.71 cm). The mean distance from the right eye to the vertical center of the frontal right earEOG electrodes was also 5.22 cm (SD = 0.71 cm). The following sections present the results and discussion of our evaluation.

### Comparison of electrode positions

#### Horizontal earEOG electrodes

Combining electrodes from the left ear reveals the best electrode pair to be L1-L4 (${\textrm{r}}_{{\textrm{EOG}}-{\textrm{L1}}-{\textrm{L4}}}=0.52, {\textrm{p}}=0.03$; ${\textrm{r}}_{{\textrm{CAM}}-{\textrm{L1}}-{\textrm{L4}}} = 0.44, {\textrm{p}}=0.03$). On the right ear, the best electrode pair was found to be R5-R8 (${\textrm{r}}_{{\textrm{EOG}}-{\textrm{R5}}-{\textrm{R8}}}=0.41, {\textrm{p}}=0.03$; ${\textrm{r}}_{{\textrm{CAM}}-{\textrm{R5}}-{\textrm{R8}}} = 0.39, {\textrm{p}}=0.04$). Combining electrodes from both ears increases the correlation further up with the best electrode pair being L8-R8 (${\textrm{r}}_{{\textrm{EOG}}-{\textrm{L8}}-{\textrm{R8}}} = 0.81, {\textrm{p}}=0.01$; ${\textrm{r}}_{{\textrm{CAM}}-{\textrm{L8}}-{\textrm{R8}}} = 0.56, {\textrm{p}}=0.02$). The results are shown in Fig. 3a for selected montages and in Supplementary Table  1 for all montages. earEOG based on electrodes at eye level have the highest correlation. Moving farther away from eye level decreases the correlation. The lowest correlation with horizontal earEOG among horizontal pairs is achieved by the montages of L3–L2, R2–R3, and L2–R2, which are the farthest away from the eye level.

The Friedman test with the gold-standard EOG as a ground truth revealed a p-value of $1.75 \times 10^{-03}$. As the p-value is below the significance level of 0.05, we can reject the null hypothesis that the electrode montages have the same correlation to the gold-standard EOG. Therefore, we performed a post-hoc analysis using the Wilcoxon signed-rank test with Bonferroni correction. The results are shown in Fig. 3b.

For the camera-based eye tracking ground truth, the Friedman test revealed a p-value of $4.15 \times 10^{-04}$. The p-value again is below the significance level of 0.05, and we can reject the null hypothesis that the electrode montages have the same correlation to the camera-based eye tracking. Again, we performed a post-hoc analysis using the Wilcoxon signed-rank test with Bonferroni correction and the results can be seen in Fig. 3c.

Notably, the test indicates, that there is no detectable difference between the best horizontal electrode montage (L8–R8) and the second-best electrode pair (L1–R1) in reference to the gold-standard EOG.

#### Vertical earEOG electrodes

The montages of L3-L7 (${\textrm{r}}_{{\textrm{EOG}}-{\textrm{L3}}-{\textrm{L7}}} = 0.3, {\textrm{p}} = 0.04$; ${\textrm{r}}_{{\textrm{CAM}}-{\textrm{L3}}-{\textrm{L7}}} = 0.31, p = 0.06$) on the left ear and R3-R7 (${\textrm{r}}_{{\textrm{EOG}}-{\textrm{R3}}-{\textrm{R7}}} = 0.32$; ${\textrm{p}} = 0.03$; ${\textrm{r}}_{{\textrm{CAM}}-{\textrm{R3}}-{\textrm{R7}}} = 0.31$; ${\textrm{p}} = 0.05$) on the right ear yield the strongest correlation (see Figure 3d for selected montages and Supplementary Table  2 for all montages). Moving farther away from the eyes decreases performance and produces much smaller correlations with L4–L5 and R4–R5 exhibiting the smallest correlation to gold-standard EOG among the tested vertical pairs.

For the vertical earEOG electrodes, the Friedman test yielded a p-value of $4.82 \times 10^{-04}$ when compared to the gold-standard EOG and a p-value of $3.76 \times 10^{-01}$ when compared to camera-based eye tracking. As the p-value for the gold-standard EOG is below the significance level of 0.05 we can reject the null hypotheses that the electrode montages have the same correlation to the gold-standard EOG. The corresponding post-hoc analysis using the Wilcoxon signed-rank test with Bonferroni correction did not reveal and significant differences between the montages and is therefore not shown.

For the camera-based eye tracking ground truth, the Friedman test revealed a p-value above the significance level of 0.05. Therefore, we cannot reject the null hypothesis that the electrode montages have the same correlation to the camera-based eye tracking system and no post-hoc analysis was performed.

#### Discussion

Signals were generally stronger when measured closer to the eyes, making the combination of electrodes from both ears advantageous as it captures more of the signal. The electrode pairs placed on the left ear generally exhibit slightly better performance compared to the gold-standard EOG, which may be due to the fact that the gold-standard EOG was recorded from the left eye.

Regarding the vertical earEOG electrodes, we found that the electrodes closest to the eyes generally were not the most effective. On both ears, the electrode pairs covering sufficient vertical distance achieve the highest correlation (L3–L7 and R3–R7). The performance of vertical earEOG electrodes further diminished as they were placed farther away from the eyes (L4–L5 and R4–R5). This suggests that a sufficient vertical distance covered by the electrodes on the ears is needed to achieve high correlation. Overall, vertical earEOG appears to be ineffective for measuring eye movements reliably.

### Saccade analysis

**Fig. 4:**
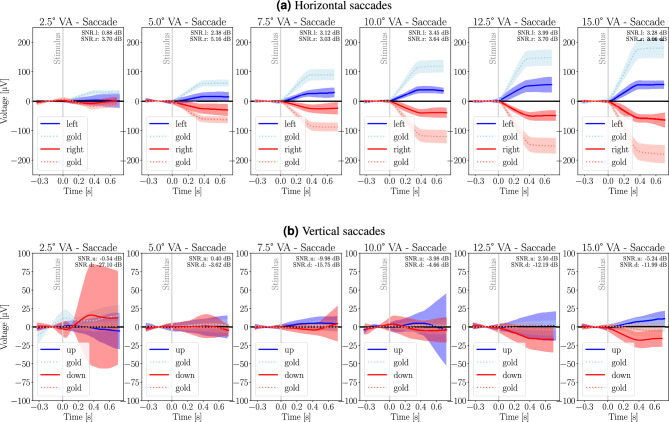
The figure displays the fixation-related EOG signals plotted for different saccade directions (left, right, up, down) and visual angles (2.5$^{\circ }$, 5$^{\circ }$, 7.5$^{\circ }$, 10$^{\circ }$, 12.5$^{\circ }$, 15$^{\circ }$). The red and blue lines represent the average earEOG signal. The dotted lines represent the average gold-standard EOG signal. The shaded area around the lines represents the standard deviation.

#### Average saccade signal analysis

To ensure the analysis was based on the most reliable data, the saccade analysis was performed exclusively with the best-performing electrode pairs identified in the preceding smooth pursuit experiment: L8-R8 for horizontal and R3-R7 for vertical saccades. Figure 4 provides insight into the average signal of the saccades for each direction and visual angle. The signals were shifted to zero based on the average voltage difference of the electrode montage before the saccade was performed to better show the relative change per angle and direction.

Upon examining the average saccade signals for each direction and visual angle, we can identify several key features and trends: (i) The EOG signals for saccades in the horizontal direction, irrespective of their amplitude, exhibit a similar waveform shape. This consistency suggests that the EOG system can reliably detect saccades in the horizontal direction and that the waveform shape is characteristic of the saccade direction; (ii) magnitude: The signal magnitude in the horizontal direction generally increases with increasing saccade amplitude, indicating a strong relationship between saccade amplitude and EOG signal magnitude; (iii) The average horizontal saccade signals are reasonably pronounced, allowing for clear identification of the saccades; (iv) vertical saccades exhibit much smaller amplitudes and are less consistent than horizontal earEOG waveforms.

#### Discussion

For horizontal saccades, the consistency in signal shape, the relationship between signal duration and amplitude, and the overall signal magnitude trends support the notion that the EOG system near the ears can effectively track eye movements across different amplitudes in the horizontal direction. As the signal for the vertical saccades is not that consistent or pronounced, this conclusion cannot be made for the vertical saccades.

### Saccade amplitudes and corresponding EOG voltage deflections

**Fig. 5:**
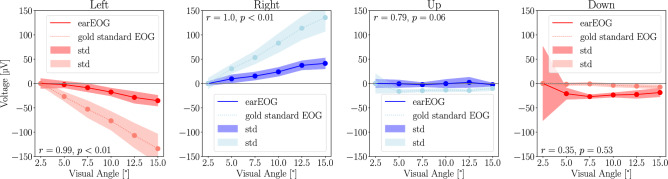
Voltage differences for each visual angle and direction of gaze when performing saccades using earEOG and gold-standard EOG. Scatter plots show the mean voltage differences for each visual angle and direction of gaze using earEOG (red/blue) and gold-standard EOG (coral/lightblue). The filled areas around the lines show the standard deviation.

The average voltage deflections for each visual angle and direction are summarized in Fig. 5. In addition, the figure shows the correlation between the absolute voltage deflections at the best earEOG positions (L8-R8 for horizontal saccades, R3–R7 for vertical saccades) and gold-standard wet electrode EOG.

#### Horizontal saccades

For horizontal saccades, the EOG voltage deflections increased with increasing saccade amplitude and shows a linear trend. The $7.5^{\circ }$ left saccade showed a deflection of −8.7 $\upmu \hbox {V}$, while the $15^{\circ }$ left saccade resulted in a deflection of −35.35 $\upmu \hbox {V}$. In the right direction, the $7.5^{\circ }$ saccade showed a 15.33 $\upmu \hbox {V}$ deflection and the $15^{\circ }$ right saccade resulted in a 41.34 $\upmu \hbox {V}$ deflection. This trend suggests that earEOG captures increasing voltage deflections with increasing saccade amplitude. For horizontal saccades, earEOG and gold-standard EOG deflections were found to be very strongly correlated at ${\textrm{r}}_{{\textrm{left}}} = 0.99$, ${\textrm{p}}_{\textrm{left}} = 0.0$ and ${\textrm{r}}_{\textrm{right}} = 1.0$, ${\textrm{p}}_{\textrm{right}} = 0.0$.

#### Vertical saccades

For vertical saccades, the EOG average voltage deflections did not show a continuous increase with increasing saccade amplitude. Upward saccades did not result in deflections, with the $7.5^{\circ }$ upward saccade showing only a -2.37 $\upmu \hbox {V}$ deflection and the $15^{\circ }$ upward saccade resulting in only a -2.4 $\upmu \hbox {V}$ deflection. Downward saccades also did not result in significant deflections, with the $7.5^{\circ }$ downward saccade showing only a -20.94 $\upmu \hbox {V}$ deflection and the $15^{\circ }$ downward saccade resulting in a -18.57 $\upmu \hbox {V}$ deflection. For vertical saccades, the correlations between earEOG and gold-standard EOG deflections were more varied compared to horizontal saccades. For upward saccades, the correlation was ${\textrm{r}}_{\textrm{up}} = 0.79$, ${\textrm{p}}_{\textrm{up}} = 0.06$. Downward saccades had even weaker correlation of ${\textrm{r}}_{\textrm{down}} = 0.35$, ${\textrm{p}}_{\textrm{down}} = 0.53$.

#### Discussion

The results demonstrate that the earEOG system reliably captures horizontal saccades with varying amplitudes but fails to consistently measure voltage changes related to vertical saccades. The voltage deflections for horizontal saccades show a more consistent, linear trend with increasing amplitude compared to vertical saccades. The correlation analysis of the absolute voltage deflections between the earEOG and gold-standard EOG provides valuable insights into the earEOG system’s performance. The high correlations for horizontal saccades indicate that the earEOG system can reliably track horizontal eye movements. On the other hand, the more varied correlations for vertical saccades suggest that the earEOG system fails to capture vertical eye movements.

### Gaze angle prediction

We performed the gaze angle prediction experiment three times: once using only the best horizontal electrode pair (L8-R8) as determined by preliminary experiments, once using all available electrode pairs and once using the gold-standard EOG. Table 1 presents the mean absolute gaze angle errors and standard deviations for the three experiments across different gaze angles ($2.5^{\circ }$, $5.0^{\circ }$, $7.5^{\circ }$, $10^{\circ }$, $12.5^{\circ }$, and $15^{\circ }$) and directions (left and right) in comparison to the Tobii eye tracker. As shown in the previous section, the earEOG system cannot reliably capture vertical eye movements; therefore, the up and down directions are excluded from this experiment. When using all available electrode pairs, the overall errors for the method using all available electrode pairs are smaller than those of the method using only the best horizontal electrode pair in both directions: Left ($3.65 ^\circ \pm 3.23 ^\circ$ vs. $4.13 ^\circ \pm 3.52 ^\circ$), right ($3.25 ^\circ \pm 2.68 ^\circ$ vs. $3.85 ^\circ \pm 3.38 ^\circ$) and combined ($3.44 ^\circ \pm 2.96 ^\circ$ vs. $3.99 ^\circ \pm 3.45 ^\circ$). However, the overall errors for the gold-standard method are even smaller: Left ($3.06 ^\circ \pm 2.44 ^\circ$ vs. $3.65 ^\circ \pm 3.23 ^\circ$), right ($2.91 ^\circ \pm 2.44 ^\circ$ vs. $3.25 ^\circ \pm 2.68 ^\circ$) and combined ($2.98 ^\circ \pm 2.44 ^\circ$ vs. $3.44 ^\circ \pm 2.96 ^\circ$).

**Fig. 6:**
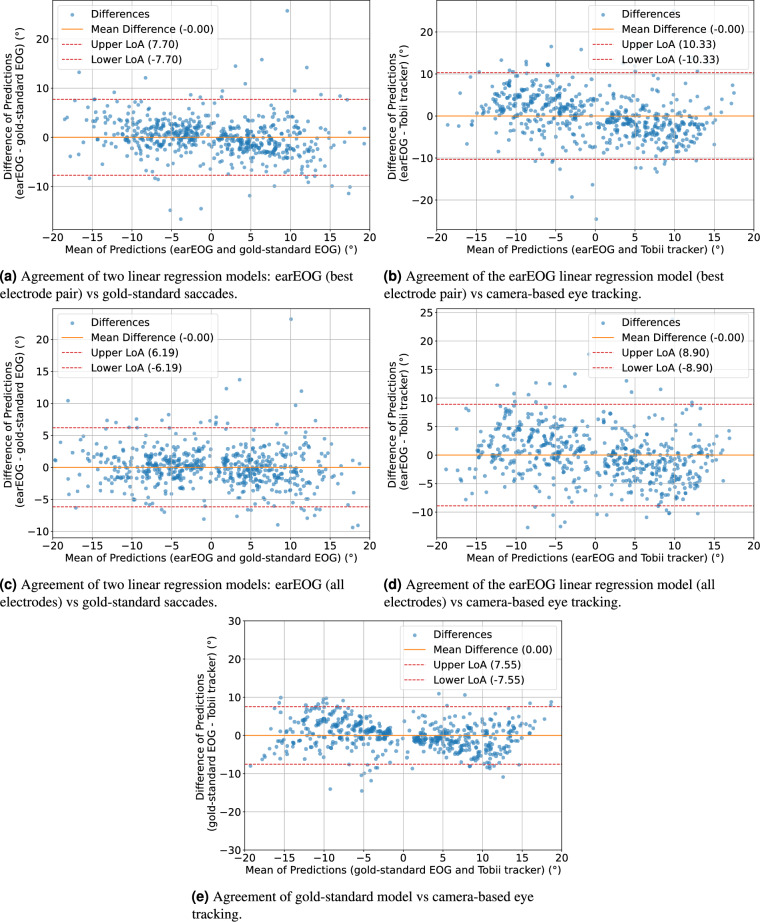
Bland–Altman plots comparing linear regression models trained on different eye-tracking signals.

To evaluate the agreement between the earEOG and the gold-standard EOG, we employ Bland–Altman plots. Figure 6a shows the Bland–Altman plot evaluating the agreement between the earEOG and the gold-standard EOG linear regression models where we use only the best horizontal electrode pair (L8-R8) to train the earEOG model while Fig. 6b shows the agreement between the earEOG and the camera-based eye tracking data. We conducted this experiment a second time using all available electrode pairs for which the experiment is shown in Fig. 6c for the agreement between the earEOG and the gold-standard EOG linear regression models and in Fig. 6d for the agreement between the earEOG linear regression model and the camera-based eye tracking data. In addition, 6e shows the agreement between the gold-standard EOG and the camera-based eye tracking data. The majority of measurements exhibit differences centered around zero, indicating no significant bias. The mean difference, also displayed on the plot, further supports this observation. Additionally, the limits of agreement (LoA) are depicted and are rarely exceeded, demonstrating consistent agreement between the two methods.

#### Discussion

The results indicate that the gold-standard method outperforms the ear-based EOG method in terms of mean absolute gaze angle errors across all gaze angles and tested directions. It can also be seen that using all available electrodes in the prediction model increases performance compared to using only the best electrode pair.

**Table 1:** Mean absolute errors (MAEs) and standard deviations were calculated for three linear regression models: one trained on ear-EOG saccade data with only the best elecrode pair, one trained on ear-EOG saccade data with all electrode pairs and the other on gold-standard EOG saccade data. Both evaluated against the Tobii eye tracking data as the ground truth.

	earEOG (best electrode pair)	earEOG (all electrode pairs)	Gold-standard EOG
Left $2.5^{\circ }$	$3.64^{\circ }$ ± $4.61^{\circ }$	$3.57^{\circ }$ ± 4.11$^{\circ }$	1.23$^{\circ }$ ± $1.22^{\circ }$
Left $5.0^{\circ }$	$2.79^{\circ }$ ± $2.26^{\circ }$	$2.32^{\circ }$ ± 2.06$^{\circ }$	1.80$^{\circ }$ ± $1.56^{\circ }$
Left $7.5^{\circ }$	$3.51^{\circ }$ ± $2.02^{\circ }$	$3.08^{\circ }$ ± 1.73$^{\circ }$	2.79$^{\circ }$ ± $1.76^{\circ }$
Left $10.0^{\circ }$	$4.25^{\circ }$ ± $3.30^{\circ }$	$4.31^{\circ }$ ± 3.63$^{\circ }$	3.53$^{\circ }$ ± $1.94^{\circ }$
Left $12.5^{\circ }$	$5.13^{\circ }$ ± $3.62^{\circ }$	$4.07^{\circ }$ ± 3.21$^{\circ }$	4.53$^{\circ }$ ± $2.90^{\circ }$
Left $15.0^{\circ }$	$5.79^{\circ }$ ± $3.84^{\circ }$	$4.92^{\circ }$ ± 3.52$^{\circ }$	5.06$^{\circ }$ ± $3.20^{\circ }$
Left total	4.13$^{\circ }$ ± 3.52$^{\circ }$	3.65$^{\circ }$ ± 3.23$^{\circ }$	3.06$^{\circ }$ ± 2.44$^{\circ }$
Right $2.5^{\circ }$	$2.07^{\circ }$ ± $2.00^{\circ }$	$1.90^{\circ }$ ± 1.73$^{\circ }$	1.66$^{\circ }$ ± $1.72^{\circ }$
Right $5.0^{\circ }$	$2.86^{\circ }$ ± $2.62^{\circ }$	$2.29^{\circ }$ ± 2.02$^{\circ }$	1.54$^{\circ }$ ± $1.22^{\circ }$
Right $7.5^{\circ }$	$3.77^{\circ }$ ± $3.57^{\circ }$	$3.24^{\circ }$ ± 2.35$^{\circ }$	2.30$^{\circ }$ ± $1.52^{\circ }$
Right $10.0^{\circ }$	$4.14^{\circ }$ ± $3.09^{\circ }$	$3.50^{\circ }$ ± 2.80$^{\circ }$	2.99$^{\circ }$ ± $2.07^{\circ }$
Right $12.5^{\circ }$	$4.44^{\circ }$ ± $4.27^{\circ }$	$3.76^{\circ }$ ± 2.91$^{\circ }$	3.75$^{\circ }$ ± $2.32^{\circ }$
Right $15.0^{\circ }$	$5.24^{\circ }$ ± $3.04^{\circ }$	$4.19^{\circ }$ ± 2.77$^{\circ }$	4.29$^{\circ }$ ± $2.63^{\circ }$
Right total	3.85$^{\circ }$ ± 3.38$^{\circ }$	3.25$^{\circ }$ ± 2.68$^{\circ }$	2.91$^{\circ }$ ± 2.44$^{\circ }$
Total	3.99$^{\circ }$ ± 3.45$^{\circ }$	3.44$^{\circ }$ ± 2.96$^{\circ }$	2.98$^{\circ }$ ± 2.44$^{\circ }$

## Discussion

In this paper, we investigated the performance of an earEOG system for tracking eye movements, specifically focusing on smooth pursuits and saccades of different directions and amplitudes. Our analysis included the correlations between the earEOG and gold-standard EOG, the examination of average saccade signals and voltage deflections, and absolute gaze angle prediction. The results of our study provide valuable insights into the potential and limitations of the earEOG system for eye movement tracking and analysis.

### Vertical vs. horizontal earEOG

Our study identified the optimal electrode pairs for measuring vertical and horizontal eye movements as L8-R8 for horizontal and R3-R7 for vertical movements. Horizontal earEOG significantly outperforms vertical earEOG, with much higher correlation values (r = 0.81, p = 0.01) for horizontal electrodes compared to vertical ones (r = 0.32, p = 0.03) when benchmarked against the gold-standard EOG system. This is likely due to the alignment of the electrodes with the eyes. In the earEOG system, the horizontal electrodes are aligned with the eye’s horizontal axis, while the vertical electrodes are offset from the vertical axis. The consistency in signal shape, duration, and magnitude trends observed in the average saccade signals and the prediction performance further support the earEOG system’s ability to effectively track horizontal eye movements. While the earEOG system reliably tracks horizontal saccades with high correlations and consistent signal patterns, vertical saccades show more variability. For the smooth pursuit task, the correlation between earEOG and gold-standard EOG were low. This is further highlighted in the saccade analysis. In the average saccade analysis, the signals are not consistent in comparison to the gold-standard and no linear trend can be observed in the up and down directions when analyzing the voltage deflections (Fig. 5). This may stem from anatomical factors, as vertical eye movements produce smaller electrical signals, even for the gold-standard measurement positions as seen in Fig. 5. These findings demonstrate the feasibility of horizontal earEOG for practical applications, whereas vertical earEOG appears to be impractical. Identified electrode pairs can be used in future research and clinical settings conducting similar research with ear-based EOG.

### Comparison to related work

Favre-Félix et al.^14^ investigated $30^{\circ }$ horizontal saccades using in-ear electrodes. They reported a voltage change of approx. 50 $\upmu \text {V}$ during saccades. While amplitude comparisons are influenced by factors like noise floor, gain or skin preparation, and direct comparisons without the same measurement equipment are difficult, earEOG based on our headphone setup achieves up to approx. 150 $\upmu \text {V}$, suggesting that saccades can be measured more reliably using the periauricular positioning of electrodes.

Manabe et al.^18^ used a related headphone setup with gaze targets that were $20^{\circ }$ apart. The electrodes were arranged around the ear, with four electrodes on each side. They reported an overall absolute gaze angle estimation error of $4.4^{\circ }$ horizontal, which closely aligns with our findings, and $8.3^{\circ }$ vertical in a study with six participants. Similar to our finding, the vertical error exceeded the horizontal error. They calibrate the model per user which increases the performance compared to our general model which is implemented as a one-fits-all approach.

Barbara et al.^27^ explored the use of EOG glasses to measure saccades for human–computer interaction. Their study demonstrated a saccade detection accuracy of 73.38% using a threshold-based classification algorithm. Notably, they distinguished between horizontal and vertical saccades using subject-specific thresholds during calibration. In our work, we did not perform saccade detection and instead, predict the angle of saccades.

### Limitations

Our study extends prior research on earEOG technology, offering new insights through a laboratory investigation with 16 participants. While this lab-study provides insight into the applicability of earEOG systems, some improvements could be made to further showcase its feasibility in the real world. Firstly, the paper assumes a center resting position for gaze, and therefore does not investigate any relative saccadic movements. As saccades play a crucial role in visual perception, this assumption may limit the generalizability of our findings. Secondly, the study does not account for head movements during saccades, assuming instead that the head is fixed in space. However, turning the head is an integral part of gaze, and this oversight may introduce bias in our results. We also acknowledge that the current setup is not optimized for all applied scenarios. This was a deliberate decision to first get a robust understanding of the fundamental potential of the approach. Identifying more applied setups (e.g. spectacle-appropriate electrode positioning) would now be possible in future work. Furthermore, while a formal analysis of inter-subject reliability was not the primary focus of this study, it represents an interesting avenue for future work. Nevertheless, the inclusion of multiple participants does provide an initial indication of the system’s reliability, as our results account for the natural signal variations observed across different individuals. Lastly, our gaze angle prediction method assumes that saccades have already been detected, and only predicts the angle of gaze. In a real-world system, the isolation of saccades would be a necessary step.

## Conclusion

In this work, we demonstrated the potential of ear-based EOG (earEOG) for measuring eye movements with varying amplitudes and directions. Our results establish the feasibility of tracking horizontal eye movements using the earEOG system, with the optimal horizontal electrode pair identified as L8-R8 (${\textrm{r}}_{\textrm{EOG}} = 0.81, p=0.01$; ${\textrm{r}}_{\textrm{CAM}} = 0.56, p=0.02$). This pair exhibited a linear relationship between the visual angle of a saccade and the corresponding voltage deflection (${\textrm{r}}_{\textrm{left}} = 0.99$, ${\textrm{p}}_{\textrm{left}} < 0.001$ and ${\textrm{r}}_{\textrm{right}} = 1.0$, ${\textrm{p}}_{\textrm{right}} < 0.001$), showcasing its potential for precise tracking.

Although the gold-standard EOG method outperforms earEOG in terms of gaze angle prediction accuracy, earEOG offers significant advantages for unobtrusive, day-to-day use. For instance, our system could pave the way for applications such as detecting dizziness or other vestibular disorders in real-world settings. However, the results indicate that earEOG is not suitable for measuring vertical eye movements. The best vertical electrode pair showed only weak correlation when users performed smooth pursuits with the best pair being R3-R7 (${\textrm{r}}_{\textrm{EOG}} = 0.32, p=0.03$; ${\textrm{r}}_{\textrm{CAM}} = 0.31, p=0.05$). Voltage deflections also were significantly less pronounced than the gold-standard EOG (${\textrm{r}}_{\textrm{up}} = 0.79$, ${\textrm{p}}_{\textrm{up}} = 0.06$ and ${\textrm{r}}_{\textrm{down}} = 0.35$, ${\textrm{p}}_{\textrm{down}} = 0.53$)

In summary, earEOG represents a promising approach for measuring horizontal eye movements, offering potential for practical applications in clinical diagnostics (e.g., dizziness detection) and gaze-based human-computer interaction. Future work should focus on enhancing system design and signal processing to further improve accuracy and explore its broader applicability, for instance by investigating combined horizontal and vertical eye movements.

## Supplementary Information

Supplementary Information.

## Data Availability

The collected data and code are available from the corresponding author upon reasonable request.
