# Supplementary material for: earEOG via periauricular electrodes to facilitate eye tracking in a natural headphone form factor
Source: Sci Rep. 2025 Sep 12;15:32437. doi: 10.1038/s41598-025-16839-z (PMC12432261; doi:10.1038/s41598-025-16839-z)
Supplement: Supplementary file 1 — Supplementary Information. [file 41598_2025_16839_MOESM1_ESM.pdf]

Supplementary Material

Horizontal Correlations

The horizontal correlations with reference to the gold-standard EOG and the camera-based eye tracking system are shown in Supplementary Table 1.

Supplementary Table 1: Correlation of horizontal eye movements with the gold-standard EOG and camera-based eye tracking system.

#	Montage	r_{EOG}	p_{EOG}	r_{CAM}	p_{CAM}
0	(L8, R8)	0.81	1.0×10^{-02}	0.56	2.0×10^{-02}
1	(L1, R8)	0.81	6.3×10^{-03}	0.57	2.0×10^{-02}
2	(L1, R1)	0.8	9.3×10^{-03}	0.56	2.2×10^{-02}
3	(L8, R1)	0.77	1.2×10^{-02}	0.55	2.3×10^{-02}
4	(L1, R7)	0.74	2.1×10^{-02}	0.52	1.8×10^{-02}
5	(L7, R8)	0.73	1.3×10^{-02}	0.53	2.6×10^{-02}
6	(L8, R7)	0.72	1.2×10^{-02}	0.5	1.6×10^{-02}
7	(L4, R1)	0.69	9.9×10^{-03}	0.51	2.2×10^{-02}
8	(L4, R8)	0.69	1.4×10^{-02}	0.51	2.2×10^{-02}
9	(L7, R1)	0.69	1.2×10^{-02}	0.52	2.5×10^{-02}
10	(L1, R5)	0.67	1.8×10^{-02}	0.49	3.2×10^{-02}
11	(L3, R1)	0.67	1.6×10^{-02}	0.48	2.9×10^{-02}
12	(L1, R3)	0.66	1.3×10^{-02}	0.48	2.7×10^{-02}
13	(L7, R7)	0.66	2.3×10^{-02}	0.49	2.6×10^{-02}
14	(L1, R2)	0.65	1.7×10^{-02}	0.48	2.4×10^{-02}
15	(L3, R8)	0.65	2.2×10^{-02}	0.47	3.5×10^{-02}
16	(L1, R4)	0.64	1.5×10^{-02}	0.49	2.6×10^{-02}
17	(L2, R1)	0.62	2.3×10^{-02}	0.47	3.0×10^{-02}
18	(L8, R5)	0.62	1.7×10^{-02}	0.46	3.1×10^{-02}
19	(L2, R8)	0.6	2.2×10^{-02}	0.46	3.0×10^{-02}
20	(L5, R1)	0.6	2.2×10^{-02}	0.47	2.5×10^{-02}
21	(L5, R8)	0.6	1.4×10^{-02}	0.47	1.9×10^{-02}
22	(L8, R3)	0.6	2.1×10^{-02}	0.45	3.1×10^{-02}
23	(L8, R2)	0.59	2.0×10^{-02}	0.45	2.7×10^{-02}
24	(L4, R7)	0.58	2.1×10^{-02}	0.46	2.9×10^{-02}
25	(L3, R7)	0.57	2.4×10^{-02}	0.43	2.2×10^{-02}
26	(L8, R4)	0.56	2.1×10^{-02}	0.45	4.0×10^{-02}
27	(L3, R3)	0.55	2.7×10^{-02}	0.41	2.4×10^{-02}
28	(L3, R2)	0.54	2.7×10^{-02}	0.42	3.5×10^{-02}
29	(L2, R7)	0.53	2.7×10^{-02}	0.43	3.2×10^{-02}
30	(L7, R3)	0.53	3.1×10^{-02}	0.43	3.3×10^{-02}
31	(L1, L4)	0.52	2.5×10^{-02}	0.44	3.1×10^{-02}
32	(L7, R2)	0.52	2.4×10^{-02}	0.43	3.4×10^{-02}
33	(L7, R5)	0.52	2.4×10^{-02}	0.43	2.9×10^{-02}
34	(L1, L5)	0.51	3.0×10^{-02}	0.43	3.6×10^{-02}
35	(L4, R3)	0.51	2.7×10^{-02}	0.41	2.8×10^{-02}
36	(L5, R7)	0.51	2.3×10^{-02}	0.42	2.8×10^{-02}
37	(L2, R2)	0.5	2.1×10^{-02}	0.42	3.6×10^{-02}
38	(L2, R3)	0.49	2.6×10^{-02}	0.4	3.7×10^{-02}
39	(L3, R4)	0.49	2.3×10^{-02}	0.41	3.3×10^{-02}
40	(L4, R2)	0.49	2.3×10^{-02}	0.41	3.0×10^{-02}
41	(L2, R4)	0.47	2.6×10^{-02}	0.42	3.1×10^{-02}
42	(L3, R5)	0.47	2.8×10^{-02}	0.39	3.4×10^{-02}
43	(L2, R5)	0.46	2.6×10^{-02}	0.4	3.2×10^{-02}
44	(L7, R4)	0.46	2.3×10^{-02}	0.42	4.0×10^{-02}
45	(L4, R5)	0.45	2.7×10^{-02}	0.4	3.9×10^{-02}
46	(L4, R4)	0.44	2.1×10^{-02}	0.41	3.4×10^{-02}
47	(R1, R5)	0.44	1.9×10^{-02}	0.39	2.9×10^{-02}
48	(L5, R2)	0.43	2.8×10^{-02}	0.39	3.3×10^{-02}
49	(L1, L7)	0.41	3.3×10^{-02}	0.39	3.4×10^{-02}
50	(L5, L8)	0.41	2.9×10^{-02}	0.39	2.9×10^{-02}
51	(L5, R3)	0.41	2.6×10^{-02}	0.37	3.4×10^{-02}
52	(R5, R8)	0.41	2.6×10^{-02}	0.39	3.8×10^{-02}
53	(L5, R5)	0.4	2.9×10^{-02}	0.38	3.0×10^{-02}
54	(R1, R4)	0.4	2.9×10^{-02}	0.38	3.7×10^{-02}
55	(L4, L8)	0.38	2.9×10^{-02}	0.4	3.3×10^{-02}
56	(R1, R7)	0.37	3.5×10^{-02}	0.4	4.2×10^{-02}
57	(R1, R3)	0.36	2.9×10^{-02}	0.38	3.9×10^{-02}
58	(L5, R4)	0.35	3.5×10^{-02}	0.38	3.7×10^{-02}
59	(R4, R8)	0.33	3.5×10^{-02}	0.36	3.5×10^{-02}
60	(L1, L3)	0.32	2.9×10^{-02}	0.38	3.2×10^{-02}
61	(R7, R8)	0.32	4.0×10^{-02}	0.39	3.7×10^{-02}
62	(L2, L5)	0.31	3.8×10^{-02}	0.33	4.7×10^{-02}
63	(L2, L4)	0.3	3.8×10^{-02}	0.35	4.3×10^{-02}
64	(L1, L8)	0.29	3.3×10^{-02}	0.35	4.8×10^{-02}
65	(L5, L7)	0.28	3.5×10^{-02}	0.34	3.8×10^{-02}
66	(L7, L8)	0.27	2.7×10^{-02}	0.35	3.9×10^{-02}
67	(L3, L5)	0.26	4.3×10^{-02}	0.31	4.5×10^{-02}
68	(R3, R8)	0.26	3.2×10^{-02}	0.36	4.1×10^{-02}
69	(R5, R7)	0.25	3.4×10^{-02}	0.33	3.9×10^{-02}
70	(L3, L4)	0.24	4.8×10^{-02}	0.31	5.1×10^{-02}
71	(R1, R8)	0.24	4.0×10^{-02}	0.33	5.1×10^{-02}
72	(R2, R4)	0.24	3.2×10^{-02}	0.33	4.4×10^{-02}
73	(R2, R5)	0.24	3.4×10^{-02}	0.32	4.7×10^{-02}
74	(L4, L7)	0.2	4.7×10^{-02}	0.34	4.7×10^{-02}
75	(R1, R2)	0.2	3.7×10^{-02}	0.34	5.2×10^{-02}
76	(L1, L2)	0.19	4.0×10^{-02}	0.32	4.4×10^{-02}
77	(R4, R7)	0.19	4.2×10^{-02}	0.31	4.3×10^{-02}
78	(L2, L7)	0.18	5.1×10^{-02}	0.32	3.4×10^{-02}
79	(R3, R5)	0.18	4.1×10^{-02}	0.29	4.9×10^{-02}
80	(R2, R3)	0.17	4.5×10^{-02}	0.32	5.4×10^{-02}
81	(L3, L8)	0.16	3.7×10^{-02}	0.35	4.2×10^{-02}
82	(L4, L5)	0.16	4.6×10^{-02}	0.32	3.6×10^{-02}
83	(R2, R7)	0.16	4.0×10^{-02}	0.32	4.4×10^{-02}
84	(R3, R4)	0.15	4.3×10^{-02}	0.3	3.5×10^{-02}
85	(L2, L3)	0.14	3.5×10^{-02}	0.32	3.7×10^{-02}
86	(L3, L7)	0.11	4.5×10^{-02}	0.28	5.1×10^{-02}
87	(R2, R8)	0.1	4.7×10^{-02}	0.32	4.8×10^{-02}
88	(R3, R7)	0.1	3.9×10^{-02}	0.29	4.4×10^{-02}
89	(R4, R5)	0.1	4.9×10^{-02}	0.31	3.3×10^{-02}
90	(L2, L8)	0.08	4.6×10^{-02}	0.31	3.8×10^{-02}

Vertical Correlations

The vertical correlations with reference to the gold-standard EOG and the camera-based eye tracking system are shown in Supplementary Table 2.

Supplementary Table 2: Correlation of vertical eye movements with the gold-standard EOG and camera-based eye tracking system.

#	Montage	r_{EOG}	p_{EOG}	r_{CAM}	p_{CAM}
0	(R3, R7)	0.32	2.9×10^{-02}	0.31	4.6×10^{-02}
1	(L4, L7)	0.31	3.2×10^{-02}	0.33	4.2×10^{-02}
2	(L4, L8)	0.31	3.6×10^{-02}	0.37	4.1×10^{-02}
3	(R3, R8)	0.31	3.5×10^{-02}	0.33	4.2×10^{-02}
4	(L2, L8)	0.3	4.2×10^{-02}	0.33	4.0×10^{-02}
5	(L3, L7)	0.3	4.3×10^{-02}	0.31	5.8×10^{-02}
6	(L3, L8)	0.3	3.7×10^{-02}	0.34	4.2×10^{-02}
7	(L7, R3)	0.3	3.8×10^{-02}	0.3	5.1×10^{-02}
8	(L8, R3)	0.29	3.9×10^{-02}	0.33	3.8×10^{-02}
9	(L1, L8)	0.28	4.0×10^{-02}	0.35	4.5×10^{-02}
10	(L2, L7)	0.28	3.5×10^{-02}	0.31	4.6×10^{-02}
11	(L3, R7)	0.26	4.6×10^{-02}	0.3	4.4×10^{-02}
12	(L3, R8)	0.26	4.5×10^{-02}	0.31	4.2×10^{-02}
13	(L4, R7)	0.26	4.0×10^{-02}	0.31	4.2×10^{-02}
14	(L1, L7)	0.25	5.4×10^{-02}	0.3	5.0×10^{-02}
15	(L4, R8)	0.25	4.2×10^{-02}	0.32	3.9×10^{-02}
16	(L7, R2)	0.25	5.4×10^{-02}	0.29	5.7×10^{-02}
17	(R2, R8)	0.25	4.5×10^{-02}	0.31	4.3×10^{-02}
18	(R4, R8)	0.25	4.4×10^{-02}	0.32	4.9×10^{-02}
19	(L2, R8)	0.24	4.6×10^{-02}	0.3	4.9×10^{-02}
20	(L8, R2)	0.24	4.7×10^{-02}	0.32	4.3×10^{-02}
21	(R2, R7)	0.24	4.8×10^{-02}	0.29	5.0×10^{-02}
22	(R4, R7)	0.24	3.7×10^{-02}	0.32	5.3×10^{-02}
23	(L2, R7)	0.23	4.9×10^{-02}	0.3	4.3×10^{-02}
24	(L7, R4)	0.23	4.1×10^{-02}	0.31	4.4×10^{-02}
25	(L8, R4)	0.23	3.8×10^{-02}	0.34	3.4×10^{-02}
26	(R3, R5)	0.23	4.6×10^{-02}	0.29	4.6×10^{-02}
27	(R1, R7)	0.22	4.6×10^{-02}	0.29	5.4×10^{-02}
28	(R1, R8)	0.22	4.5×10^{-02}	0.29	4.7×10^{-02}
29	(L5, L8)	0.21	4.4×10^{-02}	0.36	4.6×10^{-02}
30	(L8, R1)	0.21	4.6×10^{-02}	0.32	3.9×10^{-02}
31	(L1, R7)	0.2	5.4×10^{-02}	0.29	5.3×10^{-02}
32	(L1, R8)	0.2	3.9×10^{-02}	0.29	4.8×10^{-02}
33	(L5, L7)	0.2	5.0×10^{-02}	0.32	4.6×10^{-02}
34	(L7, R1)	0.2	5.0×10^{-02}	0.28	4.0×10^{-02}
35	(L7, R5)	0.2	5.0×10^{-02}	0.33	4.4×10^{-02}
36	(L2, R5)	0.19	5.3×10^{-02}	0.28	4.2×10^{-02}
37	(L3, L5)	0.19	4.4×10^{-02}	0.28	4.8×10^{-02}
38	(L3, R5)	0.19	4.6×10^{-02}	0.28	5.0×10^{-02}
39	(L5, R3)	0.19	3.4×10^{-02}	0.27	3.5×10^{-02}
40	(R5, R7)	0.19	3.9×10^{-02}	0.32	4.3×10^{-02}
41	(L1, L2)	0.18	4.5×10^{-02}	0.3	4.3×10^{-02}
42	(L8, R5)	0.18	4.3×10^{-02}	0.35	3.5×10^{-02}
43	(R4, R5)	0.18	5.3×10^{-02}	0.3	4.3×10^{-02}
44	(L1, L3)	0.17	5.0×10^{-02}	0.3	4.7×10^{-02}
45	(L2, L5)	0.17	5.8×10^{-02}	0.27	4.4×10^{-02}
46	(L5, R7)	0.17	4.2×10^{-02}	0.31	4.8×10^{-02}
47	(R1, R3)	0.17	4.3×10^{-02}	0.32	4.2×10^{-02}
48	(R2, R5)	0.17	4.8×10^{-02}	0.28	4.2×10^{-02}
49	(L1, R3)	0.16	4.7×10^{-02}	0.29	5.3×10^{-02}
50	(L4, L5)	0.16	5.6×10^{-02}	0.28	4.9×10^{-02}
51	(L5, R8)	0.16	6.0×10^{-02}	0.32	5.1×10^{-02}
52	(R5, R8)	0.16	4.2×10^{-02}	0.32	3.6×10^{-02}
53	(L2, L4)	0.15	5.4×10^{-02}	0.29	5.2×10^{-02}
54	(L2, R1)	0.15	6.2×10^{-02}	0.3	4.6×10^{-02}
55	(L3, L4)	0.15	4.6×10^{-02}	0.28	4.5×10^{-02}
56	(L5, R2)	0.15	4.8×10^{-02}	0.27	4.8×10^{-02}
57	(L3, R1)	0.14	4.3×10^{-02}	0.3	4.9×10^{-02}
58	(L4, R5)	0.14	4.9×10^{-02}	0.28	5.8×10^{-02}
59	(L5, R4)	0.14	4.1×10^{-02}	0.28	4.2×10^{-02}
60	(L1, R2)	0.13	5.1×10^{-02}	0.28	4.0×10^{-02}
61	(L4, R3)	0.13	4.5×10^{-02}	0.27	3.6×10^{-02}
62	(L1, L4)	0.12	3.8×10^{-02}	0.31	4.0×10^{-02}
63	(L4, R1)	0.12	5.0×10^{-02}	0.32	4.3×10^{-02}
64	(L4, R2)	0.12	4.7×10^{-02}	0.28	4.6×10^{-02}
65	(R1, R2)	0.12	6.0×10^{-02}	0.3	4.0×10^{-02}
66	(R1, R4)	0.12	5.3×10^{-02}	0.32	4.8×10^{-02}
67	(L1, R4)	0.11	5.0×10^{-02}	0.3	5.3×10^{-02}
68	(L2, R4)	0.11	4.7×10^{-02}	0.27	4.8×10^{-02}
69	(R1, R5)	0.11	5.1×10^{-02}	0.29	3.7×10^{-02}
70	(R3, R4)	0.11	6.3×10^{-02}	0.28	4.1×10^{-02}
71	(L1, R5)	0.1	4.8×10^{-02}	0.27	4.9×10^{-02}
72	(L3, R4)	0.1	4.9×10^{-02}	0.27	4.7×10^{-02}
73	(L7, R8)	0.1	6.2×10^{-02}	0.28	4.8×10^{-02}
74	(R2, R4)	0.1	4.6×10^{-02}	0.28	4.6×10^{-02}
75	(L2, L3)	0.09	5.1×10^{-02}	0.29	3.3×10^{-02}
76	(L7, R7)	0.09	4.6×10^{-02}	0.29	4.7×10^{-02}
77	(L1, L5)	0.08	3.8×10^{-02}	0.27	4.4×10^{-02}
78	(L3, R2)	0.08	4.8×10^{-02}	0.3	4.0×10^{-02}
79	(L3, R3)	0.08	5.3×10^{-02}	0.3	4.5×10^{-02}
80	(L5, R1)	0.08	5.5×10^{-02}	0.27	4.6×10^{-02}
81	(L8, R8)	0.08	4.7×10^{-02}	0.32	4.9×10^{-02}
82	(R7, R8)	0.08	5.5×10^{-02}	0.28	4.1×10^{-02}
83	(L1, R1)	0.07	5.4×10^{-02}	0.28	5.1×10^{-02}
84	(L2, R2)	0.07	4.8×10^{-02}	0.27	5.0×10^{-02}
85	(L2, R3)	0.07	4.6×10^{-02}	0.28	4.6×10^{-02}
86	(L4, R4)	0.07	4.6×10^{-02}	0.29	4.1×10^{-02}
87	(L7, L8)	0.07	5.9×10^{-02}	0.26	4.6×10^{-02}
88	(L8, R7)	0.07	5.2×10^{-02}	0.32	4.1×10^{-02}
89	(R2, R3)	0.07	5.0×10^{-02}	0.28	4.9×10^{-02}
90	(L5, R5)	0.06	5.9×10^{-02}	0.28	4.3×10^{-02}
